# Isolation and Functional Profiling of Seed‐Associated Bacteria From Rattlepods (*Crotalaria* spp.) With Biotechnological Potential

**DOI:** 10.1155/ijm/6560468

**Published:** 2026-05-31

**Authors:** Brenda Apiyo Odoi, Daniel M. Nthiwa, Johnstone O. Neondo, Joshua K. Muli, Peter K. Kamau, Nancy L. M. Budambula

**Affiliations:** ^1^ Department of Biological Sciences, University of Embu, Embu, Kenya, embuni.ac.ke; ^2^ Institute for Biotechnology Research, Jomo Kenyatta University of Agriculture and Technology, Nairobi, Kenya, jkuat.ac.ke; ^3^ Department of Life Sciences, South Eastern Kenya University, Kitui, Kenya, seku.ac.ke

**Keywords:** 16S rRNA, bacterial diversity, *Crotalaria*, enzymatic activity, extracellular enzymes

## Abstract

Microorganisms associated with plant seeds have recently gained increased attention due to their pivotal role in enhancing plant health through the acquisition of nutrients and plant growth promotion. Although rattlepods (*Crotalaria* spp.) are widely consumed, information regarding the bacterial diversity and functional roles within their seeds remain unexplored. This study isolated, identified, and characterized endophytic and epiphytic culturable bacteria from seeds of Kenyan rattlepods. A total of 118 seed‐associated bacterial isolates were cultured from the rattlepods (*Crotalaria* spp.), and 20 of them were identified using 16S rRNA gene sequencing. The isolates belonged to two distinct phyla: Proteobacteria and Firmicutes. The isolates belonged to four genera: nine *Bacillus,* nine *Proteus,* one *Lysinibacillus*, and one *Morganella.* BLAST analysis of partial 16S rRNA gene sequences (763‐1273 bp) showed that 19 of the 20 isolates exhibited high similarity (˃ 99%) to previously described species, whereas one isolate showed low similarity (< 97%) and may represent a putative novel species. Hydrolytic assessment of pure cultures revealed that isolates 12EpA (identified as *Proteus mirabilis*) and 4EnA1/2/1 (*Proteus vulgaris*) produced significantly (*p* < 0.05) higher protease and cellulase. Isolates 4EnA1/2/1 (*P*. *mirabilis*) and 9EnA (*P*. *mirabilis)* were the highest significant amylase producers, whereas isolates 3EnB3/3 (*Proteus* sp. [in enterobacteria]) and 10EnA 2/1 (*P*. *mirabilis*) were the highest significant lipase producers. Most seed‐associated bacteria isolated from *Crotalaria* spp. were identified as known species; however, some isolates may represent novel taxa and could serve as promising candidates for the production of biotechnologically important extracellular enzymes under mesophilic conditions.

## 1. Introduction

Rattlepod (*Crotalaria* spp.) is one of the largest genera belonging to the family Fabaceae and subfamily Aboideau, with more than 600 species distributed in subtropical and tropical Africa, and Madagascar [1, 2]. There are over 93 documented species of *Crotalaria* in Kenya distributed in different ecological zones and presenting numerous ecological and biological importance [3]. They are used as silage; used as a nutritious vegetable providing dietary vitamin A, iron, calcium, and proteins; used in controlling soil erosion; used as ornamental plants; nematode control; and as bioremediation agents [4–7]. *Crotalaria* species are widely environmentally tolerant and with adaptations to deal with metal stress and limited water supply [6; 7]. Despite their importance, the bacterial diversity and functional roles with *Crotalaria* spp. seeds remain largely unexplored. Although recent studies have highlighted the importance of seed‐associated microorganisms in enhancing plant health and promoting growth, the specific microbes of *Crotalaria* spp. seeds and their potential biotechnological applications have not been thoroughly investigated.

Plants form complex associations with a variety of structurally and functionally different microbes called holobionts that are important in promoting the resilience and fitness of the plant in fluctuating environmental conditions [8]. Among plant structures, seeds are one of the most important phases in spermatophytes (seed plants) as they initiate new crop cycles, ensure species propagation, facilitate dispersal, and promote adaptation as well as survival in new environmental habitats. Seeds also protect embryos and provide nutrients needed to resist external pressure such as dehydration [9]. They harbor rich microbial communities on their surfaces as well as in their tissues, constituting an integral part of the plant microbiome [9]. An important contributor to such communities is endophytic bacteria, which colonize plant tissues without causing any harm and establish symbiotic relationships [10]. In return for shelter and nutrients by the host plant, endophytes provide multiple benefits such as nitrogen and phosphorus supply, disease protection, improved abiotic stress tolerance, retardation of senescence, produce enzymes that are essential in the initiation of seed germination, and the synthesis of bioactive compounds, many of which are medically useful and are of biotechnological important but are often ascribed to the plants mistakenly [10, 11]. These interactions underscore the vital role of plant‐associated microbiomes in plant health, survival, and ecological success. However, research on the presence, composition, and role of seed microbiomes have been neglected for years compared with the rhizosphere and phyllosphere. Development of seed microbiomes is dependent on abiotic elements like soil type, climatic factors (including UV radiation, temperature, wind, and rainfall), and anthropogenic influences. Host genotype and plant–microbe interactions also shape the composition of the microbial communities, thus may affect the overall plant microbiome [9]. A significant amount of the data acquired on seed‐associated microbiota has been obtained through culture‐based methods with a focus on endophytic bacteria. The phylum Proteobacteria, particularly *γ*‐Proteobacteria, is the most frequently isolated group from seeds, followed by Actinobacteria, Firmicutes, and Bacteroidetes [12]. The genera *Bacillus*, *Micrococcus*, *Pantoea*, *Pseudomonas*, *Acinetobacter Staphylococcus*, and *Paenibacillus*, are commonly found in seeds. For instance, a core microbiome was found in *Crotalaria pumila* seeds for three generations [6], suggesting that these seed‐associated bacteria may be transferred to the next generation. Studies show that microorganisms can colonize both the epiphytic components of the seeds such as the husks and/or seed coat, and the endophytic components including the embryo and endosperm [9, 13]. Conditions outside and inside the seeds are not fit for most microorganisms to thrive; therefore, seed microbiome tends to exhibit special characteristics. For instance, under low nutritional conditions, these microbes can produce spores or cysts and can use phytate as a source of phosphorus [14]. Additionally, seed‐associated microbiome shows plant growth‐promoting (PGP) characteristics like siderophore synthesis, ACC (1‐aminocyclopropane‐1‐carboxylate) deaminase production, cellulase, pectinase, and protease activity, as well as motility, nitrogen fixation, phosphate solubilization, and antibiosis [15]. Sánchez‐López et al. [6] demonstrated that *C*. *pumila* seed‐associated bacteria *Methylobacterium, Sphingomonas, Staphylococcus, Brachybacterium, Kocuria, Streptomyces, Burkholderia, Variovorax, Mycobacterium,* and *Arthrobacter* isolated from *C*. *pumila* seeds showed phosphate solubilization, indole‐3‐acetic acid (IAA), and ACC‐deaminase production capacities. Seed associated microbes also produce hydrolytic enzymes that mobilize the nutrient reserves found in the seeds necessary for early seed germination, growth, and development [16]. During seed germination, amylases break down endoplasmic carbohydrates after the seeds absorb water from the soil fueling the energy needed for the embryonic shoot and root development. Additionally, the amylase activity in seeds serve as metabolic indicator of stress in seeds. The seed proteins are catalyzed by protease enzymes and broken down into peptides and amino acids that are transferred to the developing embryo, which are useful in the biosynthesis of hormones, enzymes, proteins, purines, and pyrimidines [13]. Similarly, lipases are responsible for the metabolism of triglycerols into glycerols and fatty acids, which are the source of energy for the growing embryo [11]. The specific conditions or activities exhibited by seed‐associated microorganisms are mostly useful to their host plant. The seed microbiome has been reported to produce beneficial enzymes that have biotechnological applications [15]. Some of the enzymes produced include polymer degrading enzymes cellulase, xylanases, lipases, chitinaese, proteases, and peptinases which have been biotechnologically explored for their PGP properties and other industrial processes [17] However, limited research has focused on the seed microbiome of *Crotalaria* species, its functional roles in plant health, bioremediation, biocontrol, and its biotechnological potential [6, 7, 18–21]. Therefore, the present study investigated endophytic and epiphytic bacteria isolated from the seeds of *Crotalaria* spp. for enzymes of industrial importance, specifically amylase, protease, lipase, and cellulase.

## 2. Materials and Methods

### 2.1. Experimental Design and Sample Collection

The seeds of 12 *Crotalaria* species utilized in this study were originally obtained from the *Crotalaria* project at the University of Embu [22]. The following *Crotalaria* spp. were selected: *Crotalaria brevidens, Crotalaria trichoderma, Crotalaria palida, Crotalaria laburnifolia, Crotalaria pancira, Crotalaria intermedia, Crotalaria incana, Crotalaria anagyroides, Crotalaria indicaphyla,* and *Crotalaria spectabilis.* The seeds were grown in an open field using a randomized complete block design (RCBD). Each *Crotalaria* species was grown on a single block measuring 2 m by 2 m wide and 45 m apart with the intrarow spacing of 30 cm. Following maturity, flowering, and seed production, seeds from each species were collected in triplicate in sterile disposable bags and transported to the laboratory for investigation.

### 2.2. Isolation of Endophytes From Plant Seeds

Five grams of plant seeds from each species were first washed with tap water, followed by surface sterilization through sequential immersion in 70% ethanol for 30 s, 0.1% NaOCl solution supplemented with 0.1% tween 80 for 1 min, and again in 70% ethanol for 30 s, before being thoroughly rinsed with double distilled water [23]. The final rinse water was plated onto minimal media and incubated for 14 days to ascertain the surface sterility of the seeds. The seeds were then crushed in sterile PBS using sterile mortar and pestle. The resulting aliquot was serially diluted 10‐fold and plated on modified minimal media [24], and incubated for 14 days. Morphologically distinct colonies were subcultured in nutrient agar media.

### 2.3. Isolation of Epiphytes From Plant Seed

The sterilized plant seed pods were crushed and the resulting seeds laid on minimal media [25], for 14 days. From each of the seed samples, morphologically distinct colonies were subcultured in nutrient agar medium and incubated for 14 days.

### 2.4. Morphological and Biochemical Characterization of Bacteria Isolates

Colony and cell morphology were characterized following standard microbiological protocols. The isolates were cultured on nutrient agar medium at 25°C for 24–72 h. They were subjected to gram staining and subsequently examined under a microscope. The morphological characteristics examined included colony morphology such as shape, form, elevation, margin, and pigmentation [26]. The biochemical tests performed on all the 118 isolates from *Crotalaria* spp. seeds included the MIU (motility–indole–urease) test [24], catalase production [27], citrate utilization test [28], oxidase [24], sugar fermentation tests [29], and sulfur production test [28]. Bergey′s Manual of Determinative Bacteriology was used in the probable identification of all the isolates. Twenty representative isolates were selected and analyzed for taxonomic relationship based on their 16S ribosomal RNA (rRNA) gene sequence.

### 2.5. Screening for Extracellular Enzyme P6roduction

Screening for production of extracellular enzymes was carried out by spotting each of the isolates separately onto basal media (1.0‐g K_2_HPO_4_, 0.05‐g CaCl_2_.7H_2_O, 0.1‐g MgSO_4_.7H_2_O, and 14.0‐g of agar per liter of sterile distilled water), supplemented with 5.0 g of skimmed milk, 5.0 g of starch, 5.0 g of carboxymethylcellulose, and 5.0 mL of glycerol then incubated at 25°C for 24 h. The plates were subsequently examined, and starch plates were treated with 0.6% Lugol′s solution. The existence of distinct halos surrounding the growing bacteria following staining indicated enzyme synthesis. The carboxymethylcellulose media plates were flooded with Congo red dye, then washed with 1‐M NaCl and distilled water. The appearance of clear zones around the isolates indicated positive substrate utilization [30]. The production of protease, amylase, cellulase, and lipase enzymes was indicated by observable halos around the isolates [23]. The isolates with moderate to high enzyme production potential were further characterized based on their growth response at different temperatures, NaCl concentrations, and pH levels.

### 2.6. Molecular Characterization Using Partial 16S rRNA Gene

Genomic DNA extraction was carried out on all the 20 selected isolates using the modified phenol chloroform method [31]. The extracted templates were used in the polymerase chain reaction (PCR). The 16S rRNA gene sequence was amplified by PCR using bacterial universal primers 27F (5AGAGTTTGATCTGGCTCAG‐3) and 1392R (5‐GGTTACCTTGTTACGACTT‐3). The PCR reaction mixture (25‐*μ*L total volume) contained nuclease free water (7 *μ*L), DMSO (0.75 *μ*L), reverse primer (1.25 *μ*L), forward primer (1.25 *μ*L), dNTPs (0.5 *μ*L), MgCl_2_ (0.75 *μ*L), *Taq* (12.5 *μ*L), and template (1 *μ*L). PCR amplification was performed using an Agilent Technologies SureCycler‐8800 thermocycler under the following conditions: initial denaturation (at 95°C for 2 min), followed by 18 cycles of denaturation (at 95°C for 30 s), annealing (at 50.2°C for 60 s), extension (at 72°C for 4 min), and final extension (at 72°C for 10 min) [32]. The PCR products were examined through gel electrophoresis using 1.5% (*v*/*w*) agarose gel and stored at 4°C awaiting subsequent procedures. The sequencing of the PCR products of the 20 bacterial isolates was carried out by Inqaba Biotech African′s Genomics Company, South Africa. Sequencing was done in one direction using the forward primer (27F), which targets one of the conserved regions as previously described [33]. The gene sequences were edited using Mega 11 software and the resulting sequences were matched to those in the public databases using the basic alignment search tool (BLAST) in the National Center for Biotechnology Information (NCBI) GenBank (https://www.ncbi.nlm.nih.gov/BLAST/). Sequences were submitted to the GenBank Database and accession numbers assigned (PRJNA1238184). The sequence data can be accessed by the public through this link, https://www.ncbi.nlm.nih.gov/sra/PRJNA1238184, after January 2026. The maximum likelihood alignment approach was used to transform the nucleotide differences into distance matrices [34]. The neighbor‐joining approach in Molecular Evolutionary Genetics Analysis (MEGA 11) with the Kimura 2‐parameter model incorporating gamma distribution (K2 + G) was used to account for rate variation among sites. Tree reliability was assessed through bootstrap analysis with 1000 replicates, with bootstrap values displayed at the branch nodes. *Agrobacterium tumefaciens* IAM 14141 was included as an outgroup control. The evolutionary distances are represented by the scale bar (0.10 substitutions per site) [35].

### 2.7. Data Analysis

Phenotypic profiles were visualized as a clustered heatmap in R (v4.4.1) using pheatmap (v1.0.12) and RColorBrewer. Raw data for 20 bacterial isolates across 15 categorical morphological and biochemical traits were imported from a CSV file and whitespace‐trimmed to ensure category consistency. To render nonnumeric data computationally, each trait column was converted to integer codes, preserving categorical hierarchy without imposing artificial quantitative relationships. The transposed matrix was color‐mapped using a qualitative “Paired” palette, assigning distinct colors to each unique phenotypic levels. Hierarchical clustering with Euclidean distance and complete linkage grouped isolates by phenotypic similarity and identified trait correlations.

To develop a dendrogram, clustering was performed using the Gowers distance matrix to group the different isolate characteristics using the *daisy* function in the cluster package in R software [36]. Agglomerative hierarchical clustering was then done using Ward′s method to ensure minimal variance between the clusters and a dendrogram was generated using the *hclust* function [36]. Branches and distinct cluster groups within the generated dendrogram were colored at a height of 0.7 using the dendextend package 35. Data on extracellular enzymatic activities were imported into SAS software Version 9.4 (SAS Institute, Carry, North Carolina). Analysis of variance (ANOVA) was performed by utilizing the general linear model (PROC GLM) procedure of the SAS software Version 9.4 (SAS Institute, Carry, North Carolina). Tukey′s honest significant difference (HSD) post hoc test was then used to compare the means diameters of the inhibition zones. Differences in the production of the various enzymes among the different isolates were calculated at level of *p* < 0.05. A phylogenetic tree constructed using MEGA X was employed to infer the genetic relationships of the isolates′ sequenced genes, together with the sequences from selected reference strains. A total of 20 nucleotide sequences were analyzed. The maximum composite likelihood approach was used to calculate evolutionary distances, which were expressed in terms of the number of base substitutions.

## 3. Results

### 3.1. Morphological and Cellular Characterization and Selection of Unique Isolates

**Figure 1:**
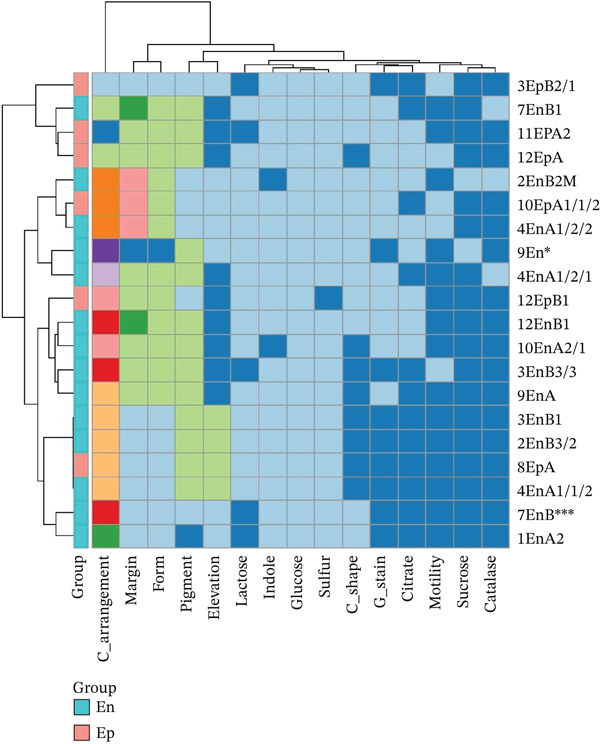
Morphological and biochemical characteristics of different bacterial isolates from the Kenyan *Crotalaria* spp. The different colors represent the different cell morphological characteristics and the biochemical characteristics. The *x*‐axis represents the morphological and biochemical characteristics, whereas the *y*‐axis represents the microbial isolates.

**Figure 2:**
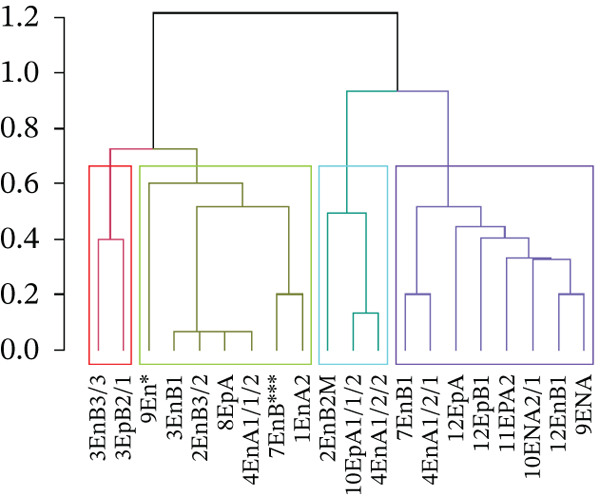
Similarity (%) of the morphological and biochemical characteristics of the selected endophytic and epiphytic bacteria isolated from the *Crotalaria* spp. in Kenya.

A total of 118 bacterial isolates were recovered from the seeds of rattlepod (*Crotalaria* spp.) based on their colony morphology (Table S1). Most of the isolates were endophytes (66) compared with epiphytes (52). Among the endophytes, 34 were gram‐positive, and 34 were gram‐negative, whereas the epiphytes comprised 34 gram‐positive and 18 gram‐negative isolates. From the 118 isolates, 20 were selected based on their distinct characteristics as revealed by the biochemical tests (Figures 1 and 2).

### 3.2. Hydrolytic Activity of the Selected Bacterial Isolates

**Figure 3:**
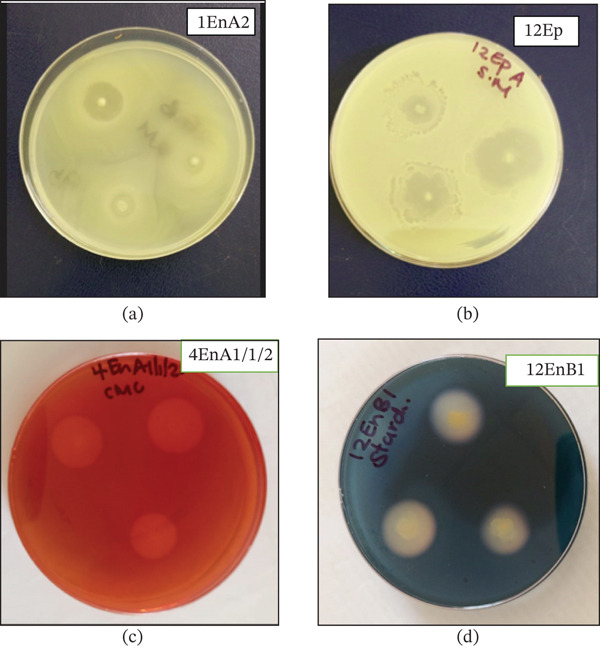
Culture plates showing positive hydrolase activities from selected sequenced bacteria isolated from the inside and on the surface of *Crotalaria* species. (a) lipase test, (b) protease test, (c) cellulase test, and (d) amylase test; the identities of the specific isolates are included on the figures. Clear zones around the bacterial growth specify positive hydrolase activity.

**Figure 4:**
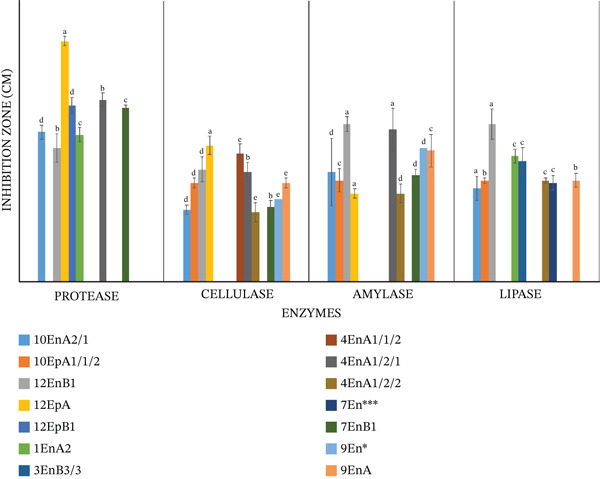
Production of extracellular enzymes by bacteria isolated from *Crotalaria* spp. in Kenya Tukey′s honest significant difference (HSD) test was utilized to compare the means of the zone of inhibition diameters. Bars (isolates) in each tested enzyme activity with the same alphabet letter denote isolate means with no statistical difference *p* = 0.05 confidence level. Values in the figures are three replicate means with standard deviations. Each horizontal category major gridline separates each enzyme, whereas the colored bars represents each of the isolates with the specific enzyme activity, with bars with the similar colors representing the same isolate. Isolates with no enzyme activity were excluded.

For hydrolytic activity, clear zones around a colony indicated the efficacy of the colony in producing a specific enzyme (Figure 3). From the selected 20 isolates, seven produced proteases, 10 produced cellulase, nine produced amylases, and eight produced lipases (Table 1). ANOVA revealed a significant difference in the production of protease (*p* = 0.001), cellulase (*p* = 0.001), amylase (*p* = 0.001), and lipase (*p* = 0.001) enzymes among the 20 studied isolates. Isolate 12EpA (*Proteus mirabilis*) was the best producer of protease and cellulase enzymes, as evidenced by producing the largest inhibition zones (3.00 and 1.7 cm for protease and cellulase, respectively), whereas isolate 12EnB1 (*Bacillus* spp.) was the best producer of amylase (1.97 cm) and lipase (1.97 cm) enzymes (Table 1). Isolates 3EnB3/3 (*Proteus* sp. [in enterobacteria]) and 7En∗∗∗ (*P*. *mirabilis*) produced lipases only (Table 1 and Figure 4).

**Table 1:** Potential hydrolytic activity of the *Crotalaria* spp. seed bacteria. All the assays documented on the table were carried out in triplicates and their means (mean ± S.E [standard error]) recorded in cm. The risk group data for each isolate was inferred from the BacDive metadatabase. The risk group levels describe the degree of hazard the bacteria pose on healthy humans. Risk Group 1 poses little to no risk to individuals and community. Risk Group 2 poses moderate risks to individuals and low risks to community.

Isolate ID	Bacterial identity	Risk group	Protease	Cellulase	Amylase	Lipase
			*M* *e* *a* *n* ± *S* *E*	*M* *e* *a* *n* ± *S* *E*	*M* *e* *a* *n* ± *S* *E*	*M* *e* *a* *n* ± *S* *E*
10EnA2/1	*Proteus mirabilis*	2	1.87 ± 0.09	0.9 ± 0.06	1.37 ± 0.42	1.17 ± 0.15
10EpA1/1/2	*Bacillus cereus*	2	0 ± 0	1.23 ± 0.07	1.27 ± 0.15	1.27 ± 0.03
12EnB1	*Bacillus* spp.	1	1.67 ± 0.18	1.4 ± 0.15	1.97 ± 0.09	1.97 ± 0.19
12EpA	*P*. *mirabilis*	2	3 ± 0.06	1.7 ± 0.16	1.1 ± 0.06	0 ± 0
12EpB1	*P*. *mirabilis*	2	2.2 ± 0.10	0 ± 0	0 ± 0	0 ± 0
1EnA2	*Lysinibacillus fusiformis*	1	1.83 ± 0.09	0 ± 0	0 ± 0	1.57 ± 0
3EnB3/3	*Proteus* sp. (in enterobacteria)	2	0 ± 0	0 ± 0	0 ± 0	1.5 ± 0.17
4EnA1/1/2	*Bacillus albus*	1	0 ± 0	1.6 ± 0.12	0 ± 0	0 ± 0
4EnA1/2/1	*Proteus vulgaris*	2	2.27 ± 0.09	1.37 ± 0.12	1.9 ± 0.26	0 ± 0
4EnA1/2/2	*Bacillus thuringiensis*	1	0 ± 0	0.87 ± 0.12	1.1 ± 0.12	1.27 ± 0.03
7En∗∗∗	*P*. *mirabilis*	1	0 ± 0	0 ± 0	0 ± 0	1.23 ± 0.09
7EnB1	*P*. *mirabilis*	2	2.17 ± 0.03	0.93 ± 0.09	1.33 ± 0.07	0 ± 0
9En∗	*Bacillus paramycoides*	2	0 ± 0	1.03 ± 0.09	1.67 ± 0.18	0 ± 0
9EnA	*P*. *mirabilis*	2	0 ± 0	1.23 ± 0.07	1.63 ± 0.20	1.27 ± 0.09

### 3.3. Characterization of the Isolates With Significant Enzyme Activities

**Figure 5:**
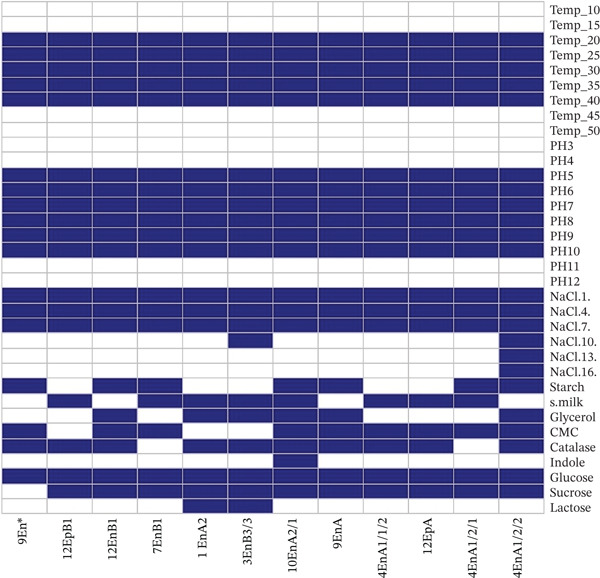
Characteristics of bacterial isolates from *Crotalaria* with significant enzyme production. The blue and white colors represent positive and negative reactions, respectively. The *x*‐axis represents the biochemical characteristics and temperature, salinity, and pH growth ranges, whereas the *y*‐axis represents the isolates that showed significant enzyme production.

The isolates which had the potential to produce significant amounts of different enzymes were further classified as presented (Figure 5). All the isolates from *Crotalaria* grew between 20°C and 40°C, pH of 5–10 and NaCl concentration of between 1% and 7% apart from isolate 4EnA1/2/2, which was able to grow at NaCl concentration of 16%. All the isolates were able to utilize glucose as their source of energy. All the isolates except 9En∗ utilized sucrose as a source of energy. Isolates 1EnA2 and 3EnB3/3 were the only isolates that utilized lactose as their source of energy.

The 20 microbial isolates were from the domain Bacteria, phyla Firmicutes, and Proteobacteria, classes Bacilli and Gamaproteobacterales, orders Caryophanales (Bacillales) and Enterobacterales, and families Morganellaceae, Enterobacteriaceae, and Bacillaceae. Among these, the *Bacillus* and *Proteus* were the most dominant genera. The *Bacilli* were *Bacillus albus, Bacillus thuringiensis, Bacillus cereus, Bacillus paramycoides*, and *Lysinibacillus fusiformis*, whereas the *Proteus* were *P. mirabilis and Proteus vulgaris.* The other bacterial species isolated from these seeds were *Morganella morganii.*

**Figure 6:**
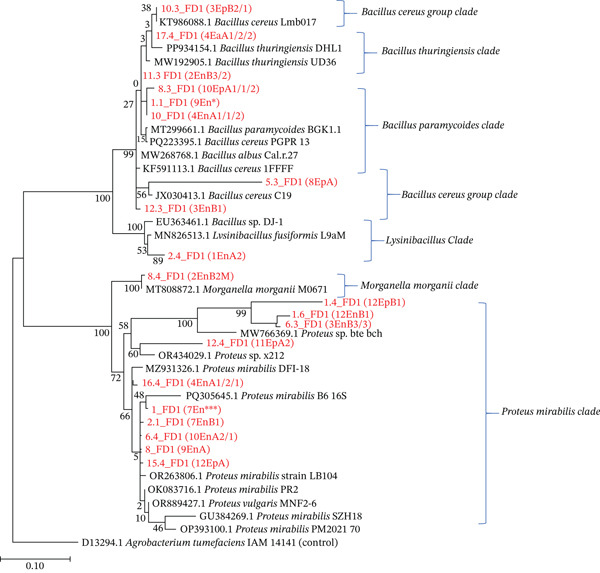
Phylogenetic relationships of epiphytic and endophytic bacteria isolated from *Crotalaria* spp. seeds based on the 16S rRNA gene sequences and the sequences from selected reference strains. The evolutionary relationship was inferred using the neighbor‐joining method. Isolates obtained from the GenBank are indicated by their isolate names and bootstrap confidence levels are shown near each node. Isolates from this study are indicated by their isolate codes. *Agrobacterium tumefaciens* IAM 14141 was included as an outgroup control. The evolutionary distances are represented by the scale bar (0.10 substitutions per site).

The affiliation of each of the 20 isolates to the closest reference bacteria strain showed that they belonged to *Bacillus*, *Morganella*, *Lysinibacillus*, and *Proteus* genera (Table 2). BLAST analysis of the partial sequences showed that 19 out of the 20 isolates showed high similarity (˃ 99%), and one had below average sequence similarity (< 97) to the *Bacillus, Lysinibacillus, Proteus*, and *Morganella* species. The *Bacillus* clade had the highest number of successfully amplified bacterial isolates distributed in the nine subclades with bootstrap values of 98 (Figure 6).

**Table 2:** Affiliation of partial sequences of 20 bacterial isolates from the *Crotalaria* seeds with 16S rRNA gene sequences in the GenBank and their assigned accession numbers as a result of BLAST.

Isolate	Species identified	Length (bp)	Similarity index (%)	Accession number
9En∗ (1.1_FD1)	*Bacillus paramycoides*	996	100%	SAMN47461948
12EpB1 (1.4_FD1)	*Proteus mirabilis*	1158	99%	SAMN47461949
7En∗∗∗ (1_FD1)	*P*. *mirabilis*	1254	100%	SAMN47461951
12EnB1 (1.6 FD1)	*Bacillus sp.*	1209	99%	SAMN47461950
7EnB1 (2.1_FD1)	*P*. *mirabilis*	1260	100%	SAMN47461952
1EnA2 (2.4_FD1)	*Lysinibacillus fusiformis*	1216	100%	SAMN47461953
8EpA (5.3_FD1)	*Bacillus cereus*	1238	100%	SAMN47461954
3EnB3/3 (6.3_FD1)	*Proteus* sp. (in enterobacteria)	1165	83%	SAMN47461955
10EnA2/1 (6.4_FD1)	*P*. *mirabilis*	1240	100%	SAMN47461956
10EpA1/1/2 (8.3_FD1)	*B*. *cereus*	1248	100%	SAMN47461957
2EnB2M (8.4_FD1)	*Morganella morganii*	1232	100%	SAMN47461958
9EnA (8_FD1)	*P*. *mirabilis*	1251	100%	SAMN47461959
3EpB2/1 (10.3_FD1)	*B*. *cereus*	1251	100%	SAMN47461960
4EnA1/1/2 (10_FD1)	*Bacillus albus*	1270	100%	SAMN47461961
2EnB3/2 (11.3_FD1)	*Bacillus thuringiensis*	1257	100%	SAMN47461962
3EnB1 (12.3_FD1)	*B*. *cereus*	763	100%	SAMN47461963
11EpA2 (12.4_FD1)	*Proteus* sp. (in enterobacteria)	1273	100%	SAMN47461964
12EpA (15.4_FD1)	*P*. *mirabilis*	1243	100%	SAMN47461965
4EnA1/2/1 (16.4_FD1)	*Proteus vulgaris*	1263	100%	SAMN47461966
4EnA1/2/2 (17.4_FD1)	*B*. *thuringiensis*	1214	100%	SAMN47461967

## 4. Discussion

This study presents a pioneer microbial analysis of the rattlepods (*Crotalaria* spp.) seeds showing the presence of a *Crotalaria* spp. seed‐associated microbial community, which has been previously unreported. These seed‐associated bacteria were isolated from the surface and the inside of sterilized seeds that were cultivated in the open field. Since the seeds used in the analysis of the endophytic bacteria were thoroughly surface sterilized, and those used in the analysis of the epiphytic associated bacteria were retrieved by first sterilizing the pods in a sterile environment, it is possible to consider the isolated bacteria as seed associated endophytes and epiphytes. An earlier study reported that apart from the potential influence on their host plants, seed associated microbes play a significant role in the plant life cycle [37]. They have been shown to contribute to seed vitality and preparation of seeds for germination. The microbes can establish microbial communities in the emerging plant, thus offering various advantages to their hosts [9; 10; 14; 15]. Most importantly, to sustain these beneficial relationships across generations, plants can utilize seeds as carriers for these microbes and transfer them to the next generations through vertical transmission [37].

The 20 out of 118 isolates screened for hydrolytic enzyme production showed that several *Crotalaria* seeds associated bacteria have the potential to produce extracellular enzymes. Although the use of culture‐based techniques for assessing microbial diversity has their limitations, they facilitate the isolation of culturable bacteria, which can be used for functional analysis or to harness their advantages in agriculture and industry [38]. Furthermore, characterizing the multifunctionality of these culturable microbes can enhance our understanding of the roles played by microbial communities that closely interact with plants, such as endophytic bacteria [38].

The phylogenetic analysis showed the relationship between the isolates and two highly distinct plant‐associated phyla [39; 40]. The sequence analysis also revealed that some of the isolates may be new species of bacteria that have never been described before; specifically, one phylotype showed less than 97% identity to published 16S rRNA genes of identified species (Table 2 and Figure 6). The 97% similarity identity index is a value that has previously been recommended as the “gold standard” of differentiating species from each other [39]. The only identified putative novel species was the *Proteus* sp. within enterobacteria. It is possible that these novel bacteria inside and outside the seeds of *Crotalaria* spp. might have coevolved with the plant and adapted to the conditions in which these plants were grown. Results from the present study therefore show that there is rich bacterial diversity possibly because of the long history of cultivation and selection under diverse climatic, edaphic, and biotic environments.

Most of the isolates were gram‐positive with Firmicutes being the dominant bacterial phylum (Tables 1 and 2). Firmicutes, especially the *Bacillus* species, have been previously reported to be endospore‐forming ubiquitous bacteria in seeds [10, 40]. The main feature that supports the existence of *Bacillus* in and on seeds is their ability to form endospores. Therefore, they can withstand the adverse changes occurring inside the seed such as the high osmotic pressure, accumulation of starch, and desiccation [10, 41] thereby providing a mechanism for them to stay dormant and survive within the desiccated *Crotalaria* spp. seeds.

The *Crotalaria* spp. seeds were also harvested during the hotter and drier month of March, which experiences an annual average temperature of 27.3°C in Embu County, Kenya. This supports the presence of *Bacillus* on the surface of these seeds as they are known to survive in unfavorable conditions and remain dormant until germination when favorable conditions return.

The isolates mainly belonged to the family Enterobacteriaceae, which include enteric bacteria that are major inhabitants of nonamended sites as well as virgin soil and agricultural soil [42] (Tables 1 and 2). Previous studies have shown that enteric bacteria are frequently found in plant parts because of their high frequency in soil [43]. The genus *Proteus* is a plant, animal, and human‐specific pathogen; according to studies, an increasing number of these genera have been shown to have positive effects on a variety of plants [44, 45]. *P*. *mirabilis, P*. *vulgaris*, and *M*. *morganii*, members of the Enterobacteriaceae family, were isolated from healthy *Crotalaria* spp. seeds for the current study.

The bacteria isolated in this study were able to produce hydrolytic enzymes such as amylases, proteases, cellulases, and lipases at an optimum temperature of 25°C and at pH 7 (Figure 5). Screening of microorganisms for their ability to produce hydrolytic enzymes promotes the discovery of novel enzymes that are important for specific use in industry such as cosmetics, food, detergents, and bioremediation as well as understanding their ecological roles to their hosts [45]. Cellulase‐producing bacteria have been isolated from plants [38]. This enzymatic activity is closely linked to the ability of an isolate to penetrate and proliferate within plant tissues. Enzymes such as cellulases, xylanases, pectinases, and endoglucanases alter the plant cell wall, thereby facilitating the entry and colonization by endophytes and supporting mutualistic plant‐microbe relationships [38]. Hydrolytic enzymes produced by seed‐associated microbes mobilize the nutrient reserves present in the seed, promoting early seed development and colonization [16]. During water uptake, cellulases degrade endoplasmic carbohydrates to provide a supply of energy for shoot and root development while also serving as metabolic indicators for seed stress [11]. Proteases catalyze seed proteins into amino acids and peptides that are essential in embryo development and biosynthesis of enzymes, hormones, purines, pyrimidines, and proteins, and lipases convert triglycerides into fatty acids and glycerol that provide energy to the developing plant [11]. Enzymes play a crucial role in the detergent industry and are a sustainable alternative to chemical additives. They are cheaper and less harmful to the environment and have specific cleaning actions that enhance stain removal efficiency. They can also be used at lower temperatures compared with chemical additives [46, 47]. Some consumers prefer to use cold water and mild conditions to wash their clothes, requiring that detergents be able to work under these conditions [48]. In the present study, enzymes were produced at room temperature (25°C) and at neutral pH (7.0); therefore, they have potential use in the production of gentle detergents which help with the maintenance of the integrity of delicate garments and skin while working at room temperature.

Proteases have been used for a long time in the food and feed industry, waste management, tanning of leather, bating of hides, and dehairing as an alternative to harmful chemicals [49]. In the current study, skimmed milk was used as the sole substrate for protease activity quantification of the isolates. From the isolates assayed, seven showed positive protease activity, and they were from the *Proteus, Lysinibacillus,* and *Bacillus* species. The genus *Proteus* was the best producer of proteases in this study. These bacteria are gram‐negative proteolytic rods [50]. Other studies have also isolated *Proteus* from different parts of the plants such as the rhizosphere [51], stem, roots, and leaves [44], and they perform different functions. Some extracellular proteases assist microbes in colonizing plant roots, whereas some proteases secreted by specific *Bacillus* spp. are toxic to nematodes through cuticle degradation activity [38].

In the current study, the genera *Bacillus* and *Proteus* were able to hydrolyze both the starch and carboxylmethlcellulose. Apart from its uses in detergents, bacterial amylases are useful in the food and textile industry as well as paper and fuel production [52]. Extracellular proteases and cellulases are also essential in the formation of peptide and polysaccharide‐rich biofilms that help in the establishment of microbial community and promote attachment to host cells such as rhizosphere surface or inside plants [53]. The *Bacillus* isolated from plant seeds have been found to be amylase producers [54]. The majority of bacterial isolates of *Bacilllus* and *Proteus* genera were also found to produce cellulases. Cellulase production by *B*. *cereus, Bacillus vulgaris* and *B. paramycoides* are well described in literature, and some of them have extensively been characterized [55–57]. The present study determined that *P. mirabilis* have the potential to produce significant amounts of cellulase. In concurrence with our results, [44] isolated and characterized *P. mirabilis* with the cellulytic activities from tomato plants. Although *P. mirabilis* with cellulytic activity have been previously isolated from different plant parts, those from the seeds have not been reported until now. Studies on the lipolytic enzyme production by different bacteria are few; however, in the present study, several isolates produced extracellular lipases: *L*. *fusiformis, B. cereus, Proteus* spp.*, Bacillus* spp.,*P. mirabilis*, and *B. thuringiensis* (Table 1). [58] isolated extracellular lipoprotein lipase producing *L. fusiformis* from dairy industry waste. [59] also isolated *B. paramycoides* from *Brassica napus* that had the ability to produce lipases that were essential in PGP. [60] found that *P. mirabilis* has the capacity to produce lipases in a wide range of temperatures and pH due to their varied biological activities. In natural environments, *Proteus* spp. exhibit exceptional metabolic characteristics, for example, *P. mirabilis* strains isolated from Chinese coastal seawater and fish processing plant effluents in India were characterized as heterotrophic nitrifiers as they effectively removed ammonia (NH_4_^+^) ions by oxidation [61, 62]. The lipolytic ability of *Proteus* species could be useful as a sole degrader of oil hydrocarbons in soil and other natural environments. *P. mirabilis* isolated from the rhizosphere of legumes grown on crude oil polluted soils in Kaduna, Nigeria were characterized as the most active crude oil degraders [63], similarly, *P.mirabilis* isolated from freshly killed fish from Niger Delta were able to utilize Bonny light crude oil, diesel, and kerosene, generating organic acids [64]. Most of the *P. mirabilis* that have been previously isolated and found to have lipolytic abilities were mostly from clinical samples and hydrocarbons polluted environments, making it very difficult to make comparisons. Therefore, the current study is an important study on the lipolytic ability of plant isolated *P. mirabilis.*

The presence of *Bacillus* as one of the main occurring seed associated bacteria in the present study could also present an important ecological significance to the host plant. Several studies have indicated that seed associated *Bacillus* have antagonistic properties against phytopathogens. *Bacillus amyloloquefaciens* isolated from *Hedera helix* seeds exhibited strong hormonal growth effects and protected the host plant from diseases caused by *Alternaria tenuissima* [65]. Similarly, four out of the six effective antagonists identified against tomato pathogens from tomato seeds belonged to the genus *Bacillus* [66]. In another study, approximately 70% (118 out of 169) of bacterial seed endophytes demonstrated antagonistic activity against five different phytopathogens affecting cucurbit vegetables [38]. *Bacillus* species produce lipopeptides, which aid in the hydrolysis of fungal hyphal membranes leading to nutrient leakage, thus reducing fungal virulence [67]. *Bacillus* also have indirect mechanisms in phytopathogen control through the stimulation of the ethylene/jasmonate–based defense response, necrotrophic microbes, and a salicylic acid–based defense against biotrophic microorganisms [67]. *Bacillus* have PGP properties. They have been shown to produce auxins specifically IAA, which are responsible for cell elongation leading to larger fruit size, AAC‐deaminase which is essential in relieving the inhibitory effect of ethylene on root growth [68]. Nitrogen and phosphates are the most limiting nutrients in plant growth, but seed‐associated *Bacillus* have the potential to carry out nitrogen fixation and/or scavenging and phosphate solubilization making them bioavailable to plants [38].

Members of the genus *Proteus* were the most significant producers of cellulase, protease, amylase, and lipase in the present study. Bacteria from the genus *Proteus* are mostly opportunistic pathogens that cause various infections in humans, including urinary tract infections, wounds, and respiratory tract, skin, eye, ear, nose, and throat infections [69]. This characteristic of the *Proteus* species provides a challenge for its use in the biotechnology industry as a whole organism, providing the need for safer biotechnological modifications or large‐scale enzyme production in controlled and safer environments.

## 5. Conclusions

The data reported in this study demonstrated that *Crotalaria* spp. seeds harbor endophytic and epiphytic bacteria, some of which are novel and have the potential to produce biotechnologically important enzymes. Furthermore, a relatively high proportion of the endophytic and epiphytic bacteria isolated from the *Crotalaria* spp. were putatively novel. The present study isolated bacteria from the surface and inside of seeds of *Crotalaria* spp. Although this study was carried out in line with the identification or prospecting for bacteria that can be found on the surface and inside the *Crotalaria* spp. and their potential biotechnological importance, the extracellular enzymes can be further evaluated in terms of their relationship with their hosts and their ecological and agricultural significance.

## Author Contributions

**Conceptualization:** Joshua K. Muli, Daniel M. Nthiwa, and Nancy L.M. Budambula; **data curation:** Brenda Apiyo Odoi; **data analysis:** Brenda Apiyo Odoi, Joshua K. Muli, and Johnstone O. Neondo; **funding acquisition:** Johnstone O. Neondo, Peter K. Kamau, and Nancy L.M. Budambula; **investigation:** Brenda Apiyo Odoi and Nancy L.M. Budambula; **methodology:** Brenda Apiyo Odoi, Joshua K. Muli, and Daniel M. Nthiwa; **drafting the original draft:** Brenda Apiyo Odoi; **draft review and editing:** Nancy L.M. Budambula, Peter K. Kamau, Joshua K. Muli, Daniel M. Nthiwa, and Johnstone O. Neondo.

## Funding

This work was funded by the National Research Fund, Kenya. The grant had no number.

## Disclosure

Brenda Apiyo Odoi, Nancy L.M. Budambula, Peter K. Kamau, Joshua K. Muli, Daniel M. Nthiwa, and Johnstone O. Neondo approved the final draft. No generative AI software was used in this work.

## Conflicts of Interest

The authors declare no conflicts of interest.

## Supporting information


**Supporting Information** Additional supporting information can be found online in the Supporting Information section. **Supporting Information.** Table S1: Morphological and biochemical characterization of seed‐associated bacterial isolates isolated from endophytic (En) and epiphytic (Ep) fractions of different *Crotalaria* species.

## Data Availability

All the data generated or analyzed from this study are included in this published article. Sequences were submitted to the GenBank Database and accession numbers assigned (PRJNA1238184). The sequences data can be accessed by the public through this link: https://www.ncbi.nlm.nih.gov/sra/PRJNA1238184 after January 2026.
